# Beneficial Effects of Ethanol Consumption on Insulin Resistance Are Only Applicable to Subjects Without Obesity or Insulin Resistance; Drinking is not Necessarily a Remedy for Metabolic Syndrome

**DOI:** 10.3390/ijerph8073019

**Published:** 2011-07-21

**Authors:** Hirokazu Yokoyama

**Affiliations:** Health Center, Keio University, 35 Shinanomachi, Shinjukuku Tokyo 160-8582, Japan; E-Mail: wdphq489@yahoo.co.jp; Tel.: +81-3-5363-3534; Fax: +81-3-5363-3535

**Keywords:** ethanol, insulin resistance, obesity, metabolic syndrome, adiponectin

## Abstract

Although moderate drinking has been shown to lower insulin resistance levels, it is still unclear whether alcoholic beverages could be remedies for insulin resistance. To elucidate this, the correlation between levels of ethanol consumption and insulin resistance were cross-sectionally examined in 371 non-diabetic male Japanese workers. Multiple regression analysis demonstrated that the ethanol consumption level was inversely correlated with the insulin resistance level assessed by homeostatic model assessment (HOMA-IR, p = 0.0014), the serum insulin level (p = 0.0007), and pancreatic β-cell function, also assessed by HOMA (HOMA-β, p = 0.0002), independently from age, body mass index (BMI), and blood pressure, liver function tests, and lipid profiles status, as well as serum adiponectin. The correlations were true in subjects with normal BMIs (up to 25.0 kg/m^2^, n = 301) or normal HOMA-IR (up to 2.0 μIU·mg/μL·dL n = 337), whereas all of them were non-significant in those with excessive BMIs (n = 70) or in those with HOMA-IR of more than 2.0 (n = 34). Although it is still unclear whether the reductions of these parameters by ethanol consumption are truly due to the improvement of insulin resistance, at least, these effects are not applicable to subjects with obesity and/or insulin resistance. Thus, alcoholic beverages could not be remedies for insulin resistance or metabolic syndrome.

## 1. Introduction

The issue of the ever-increasing incidence of metabolic syndrome causing lethal cardiovascular events, is now a critical menace to public health and medical economics, especially in developed countries [1]. In that insulin resistance, practically, following hyperinsulinemia, should be its pivotal component [2], improvement of subjects’ insulin resistance level and hyperinsulinemia must be an ultimate strategy for metabolic syndrome. Based on this, a recently prevailing view that a mild to moderate level of ethanol consumption reduces the level of insulin and/or insulin resistance is noteworthy [3–10]. Consistently, consuming ethanol at acceptable levels has been shown to be associated with lower risks of type 2 diabetes [11–13]. Analogously, moderate alcohol consumption has been shown to reduce risks of metabolic syndrome [14] regardless of the types of alcoholic beverage [15], cardiovascular events [16,17], and mortality [18–20]. In spite of these multiple lines of evidence, it is still obscure whether alcoholic beverages could be remedies for subjects suffering from metabolic syndrome. The aim of this study was to know whether physicians should prescribe a moderate drinking habit for subjects with insulin resistant.

## 2. Experimental Section

### 2.1. Subjects

This study was approved by the Ethics Committee of the Health Center, Keio University, based on the ethics guidelines of the 1975 Declaration of Helsinki, revised in 1983. Subjects were active male workers in a Japanese corporation who did not have health problems limiting their duties. They underwent health checkups in our office in 2004. First, 505 males, who consented to the use of their data for this study, were enrolled. Diabetic subjects who received the administration of medication(s) for diabetes mellitus or subjects whose levels of FBS and/or HbA1C exceeded 100 mg/dL and/or 5.5%, respectively, were excluded. Moreover, subjects who could not complete the health checkups including questionnaires listed below were excluded. Finally, 371 males, aged 30 to 65 years, were subjected. In the health checkups, present and past illnesses were asked for subjects routinely.

### 2.2. Measurements

The body mass index (BMI: kg/m^2^) was determined from the weight and height of each subject. According to the consensus in Japan, a BMI of more than 25.0 kg/m^2^ was designated as obesity. Blood platelet (PLT) level, serum levels of aspartate aminotransferase (AST), alanine aminotransferase (ALT), gamma glutamyl transpeptidase (γ GTP), fasting blood glucose (FBS), hemoglobin A1C (Hb-A1C), triglycerides (TG), low-density lipoprotein cholesterol (LDLC), and high-density lipoprotein cholesterol (HDLC) were routinely measured in each subject. Serum levels of fasting insulin and high-molecular adiponectin were examined by a commercial laboratory (SRL Inc., Tokyo, Japan) by radio-immune assays. Employing homeostatic model assessment (HOMA), levels of insulin resistant (HOMA-IR) and pancreatic β-cell function (HOMA-β) were estimated according to the established formulas [21]. Using questionnaires, an averaged amount of daily ethanol consumption and an averaged number of drinking days per week were ascertained by asking each subject. Based on the replies, an averaged weekly ethanol consumption level was determined for each subject.

### 2.3. Statistical Analysis

The differences between the two groups were non-parametrically assessed employing Mann Whitney’s U-tests independently from their distribution appearances. The correlations between multiple factors were assessed by multiple regression analyses. To perform parametric analyses, values of parameters were log-transformed to make the distributions close to normal, when they showed skewed appearances. The difference in prevalence between two groups was estimated by χ^2^ analysis followed by Fisher’s test. All statistical analyses were performed using StatView^®^ (ver. 5.0, SAS Institute Inc., NC, USA) on a personal computer (FMV-c8229, Fujitsu Co., Tokyo, Japan). P-values less than 0.05 were considered to indicate significance.

## 3. Results and Discussion

### 3.1. Subjects’ Profiles

Subjects’ profiles are shown in Table 1. In the present study, subjects’ insulin resistance statuses were evaluated by the HOMA model. Since the model may not be applicable for diabetic subjects, especially those who receive medications for diabetes [21], they were excluded from the present study. In the present population, the lowest PLT level was 119,000/μL, namely subjects who were apparently suffering from progressed liver cirrhosis were not included. Information on subjects’ present and past illnesses indicated that there were no subjects suffering from chronic alcoholic pancreatitis in the present population. There were 33 subjects whose daily ethanol consumption levels were more than 50 g/day. All of them consistently consumed ethanol 3–7 times per week and there were no apparent binge drinkers. There were 70 obese subjects whose BMIs were more than 25 kg/m^2^ in the present population. Their levels of HOMA-IR and liver function tests were poorer compared to those in non-obese subjects. The prevalence of subjects whose HOMA-IRs were more than 2.0 was 21 (30.0 %) and 15 (5.0%) in obese subjects and non-obese subjects, respectively (p < 0.0001).

### 3.2. Correlation between Levels of Ethanol Consumption and HOMA-IR and HOMA-β

The correlations between levels of ethanol consumption and HOMA-IR, HOMA-β, serum insulin, or FBS were examined employing multiple regression analyses. It is well-known that subjects’ age, BMI, lipid profiles [22], and their statuses of hypertension [23] and fatty liver [24] are relevant to insulin resistance. In addition, adiponectin, a molecule derived from adipocytes, has been implicated in the development of insulin resistance [25]. Thus, these factors were arrayed as dependent factors together with the level of ethanol consumption in the present multiple regression analysis models.

They demonstrated that the ethanol consumption level was inversely correlated with levels of HOMA-IR, HOMA-β, or serum insulin independently from the other dependent factors arrayed when all subjects were examined (Table 2). These observations are in keeping with former publications emphasizing the opinion that ethanol consumption has a potential to improve insulin resistance [3–10]. Since drinking patterns, especially binge drinking [26], types of alcoholic beverage [27,28], and gender [27,28] have been reported to be related to risks of metabolic syndrome and/or diabetes, they may also influence HOMA-IR and HOMA-β. However, since binge drinkers and females were not included in the present population, we did not study these points at this time. Furthermore, since we did not specified types of alcoholic beverages in the present study, we could not have opinions on effects of this factor at this time.

Recently, the mechanisms of how insulin resistance develops have been clarified. Usually, it is associated with the development of obesity. As obesity progresses, surplus lipids are stored in adipocytes, resulting in their enlargement [29]. From such adipocytes, the secretion of free fatty acids increases [30], meanwhile that of adiponectin decreases [25]. The former accelerates free fatty acid uptake by peripheral tissues including the skeletal muscle and liver [31], and the latter disturbs the activation of insulin receptors and consequent glucose influx into peripheral cells [32]. Both lead to the development of insulin resistance. Furthermore, the accumulation of macrophages in the visceral fat tissue [33], provably, various cytokines including TNF derived from them [34], also cause insulin resistance. They suitably explain why insulin resistance often develops in obese subjects.

The following are plausible explanations of how ethanol improves insulin resistance levels. Acetate, an intermediate of ethanol metabolism, suppresses free fatty acid release from adpiocytes [35]. Moreover, ethanol administration up-regulates some anti-inflammatory genes such as IL-10 and adrenergic beta receptor kinase 1 [36]. They may contribute to the inhibition of inflammation in visceral fat tissue. In addition, ethanol consumption has been shown to increase the serum adiponectin level [37,38]. These mechanisms may lie behind the inverse correlation between levels of ethanol consumption and HOMA-I shown here. However, before drawing the conclusion that ethanol consumption improves insulin resistance and truly exhibits beneficial effects against the development of diabetes, minute investigations seem to be needed. Indeed, there are also several lines of evidence indicating that drinking does not affect [39] or rather increases the prevalence of diabetes [28,40].

The present study also demonstrated the inverse correlation between levels of ethanol consumption and HOMA-β. This may be attributed to the improvement of insulin resistance by ethanol consumption. However, ethanol suppresses insulin secretion from pancreatic β-cells [41], probably via the down-regulation of glucokinase in them [42]. In addition, pancreatic cirrhosis, which sometimes seen in alcoholics, may also hamper insulin secretion from the pancreas. In that the former events do occur at an ethanol concentration of much higher than the physiological level [43], we believe that they were not involved in the present observations. Furthermore, in that this population did not contain subjects suffering from diabetes and chronic alcoholic pancreatitis, two major complications of pancreatic cirrhosis, it is most likely that those who were suffering from severe pancreatic cirrhosis were not included in the present subjects. However, we do not know whether subjects with alcoholic cirrhosis in early stages exist in the present population. To have definitive view that the inverse correlations between levels of ethanol consumption and HOMA-β are truly due to the reduction of insulin resistance, further in–depth evaluations of the status of the pancreas should be necessary.

Ethanol consumption leads to an increase in the NADH/NAD ratio *in vivo.* This change, the so-called redox shift [44], attenuates gluconeogenesis in the liver, resulting in the reduction of glucose efflux from it [45]. It is conceivable that this lightens the loads of pancreatic β-cells, *i.e.*, reduces insulin release from them. Since the serum insulin level must become lower under such a condition, not only the HOMA-β level but also the HOMA-IR level must decline. In that the rest of the pancreatic β-cells must contribute to the prevention of the development of diabetes [46], it is fully conceivable that this effect of ethanol contributes to the prevention of development of diabetes. However, the reduction of the HOMA-IR via this mechanism is clearly not related to the improvement of insulin sensitivity. The updated version of the HOMA model (HOMA2) is now available to calculate true HOMA-IR, in which the effect of hepatic glucose efflux is adjusted [47]. However, it was not applicable for the present study, since the model is set to be valid when the serum insulin level is between 2.9–57.6 μIU/μL [48] at this time and the present population included a large number of subjects whose insulin levels were lower than the limit. It must be noted that they were not diabetic despite having such serum insulin levels and the lower limit of the normal range of serum insulin for Japanese is presently set as 1.84 μIU/μL [49]. Observations of the relationship between ethanol consumption and HOMA-IR estimated by the HOMA2 model must be helpful for knowing the mechanisms which lies behind the correlation between levels of ethanol consumption and HOMA-IR.

### 3.3. Effects of Insulin Resistance or Obesity on Correlation between Levels of Ethanol Consumption and HOMA-IR and HOMA-β

To investigate whether alcoholic beverages could be effective remedies for insulin resistance or metabolic syndrome, correlations among levels of ethanol consumption and HOMA-IR, HOMA-β, serum insulin, or FBS were selectively examined in subjects with obesity or insulin resistance employing the multiple regression analysis models described above. Correlations between ethanol consumption and HOMA-IR, HOMA-β, and serum insulin were true in non-obese subjects whose BMIs were 25 kg/m^2^ or less (Table 3) or in non-insulin resistant subjects whose HOMA-IR levels were 2.0 μIU·mg/μL·dL or less (Table 4). However, they were non-significant in subjects with obesity (Table 3) or insulin resistance (Table 4). These observations suggest that drinking neither has potential to improve insulin resistance in subjects with obesity and/or insulin resistance nor that to improve their morbid conditions.

Fueki *et al.* reported that a drinking habit was inversely associated with HOMA-IR levels regardless of subjects’ BMIs in Japanese [50]. However, their findings, in which drinking levels were expressed as a frequency of ethanol consumption, seem not to be comparable to ours, in which they were expressed as the amount of consumed ethanol.

At this time, we do not know the exact reason why such effects of ethanol on HOMA-IR, HOMA-β, and serum insulin are canceled out in subjects with obesity and/or insulin resistance. It is postulated that levels of HOMA-IR and HOMA-β in drinkers with obesity and/or insulin resistance are determined by counterbalances between their aggravations due to obesity and/or insulin resistance and their improvements due to drinking. If the former are overwhelmingly stronger than the latter, or the latter are canceled by the former, the present observations could be suitably explained.

Recently, the importance of hepatic insulin extraction has been emphasized as a determining factor of insulin resistance and pancreatic β-cell function [51] and a current trend is to evaluate insulin resistance with adjustment for peripheral and hepatic insulin resistance [52]. To estimate them respectively, *i.e.*, to have the disposition index, euglycaemic-hyperinsulinemic clamp technique in combination with the intravenous glucose tolerance test [53] or an alternative method employing the oral glucose tolerance test [54] are available. Because of their technical complexities, they were not applicable for the present study; however, the latest study demonstrated that liver function tests could be reliable markers of hepatic insulin resistance [55]. Namely, increases in AST, ALT, and γGTP reflect a change for the worse of hepatic insulin resistance. Thus, the present findings that levels of these liver function tests in obese subjects were higher than those in non-obese subjects (Table 1) indicate that the former are suffering from hepatic insulin resistance. Ethanol consumption causes diverse kinds of stress in various organs including the liver. Even if they are tolerable in the normal liver, they may synergistically worsen liver damage and consequent hepatic insulin resistance in the liver of a subject with hepatic insulin resistance, namely that with obesity and/or insulin resistance. This could also provide an explanation why beneficial effects of ethanol consumption on HOMA-IR and HOMA-β are canceled out in such a subject.

Insulin resistance develops in a patient suffering from liver cirrhosis or its prodromal stage, *i.e.*, liver fibrosis. Excessive ethanol consumption and obesity may make a status of liver fibrosis synergistically worse, causing the development of insulin resistance. This also accords with the present observation that beneficial effects of ethanol consumption on HOMA-IR and HOMA-β are canceled out in obese subjects. Although there were no patients apparently suffering from progressed liver cirrhosis in the present population, patients suffering from liver fibrosis in various stages may exist. Strictly speaking, to known correlations between levels of ethanol consumption and HOMA-IR and HOMA-β, the parameter(s) reflecting the development of liver fibrosis which cannot be assessed by routine liver function tests should be also adjusted.

It is well-established that glucose efflux from the liver is regulated by glucagon/insulin balance. In the hepatic insulin-resistant state, the effects of glucagon become predominant. This increases glucose efflux from the liver, resulting in an increase in loads of pancreatic β-cells [56]. Thus, it is conceivable that an above mentioned the effect of ethanol consumption, *i.e.*, a decreases in glucose efflux from the liver via the redox shift, is canceled out in subjects in a hepatic insulin-resistant state. This could also account for the fact that beneficial effects of ethanol consumption on glucose/insulin dynamics are canceled out in obese and/or insulin-resistant subjects.

The present multiple regression analysis models failed to show significant correlations between levels of ethanol consumption and serum adiponectin (Table 5). The findings suggest that mechanisms irrelevant to adiponectin lie behind the beneficial effects of ethanol consumption on HOMA-IR and HOMA-β seen in the present population. The finding apparently contradicts the former data shown by Sierksma *et al.*, demonstrating the beneficial effects of ethanol on serum adiponectin [35]. However, differences in the characteristics of subjects may account for this contradiction: Sieksma *et al.*, studied subjects with insulin resistance, but we studied those without it.

A recently prevailing view is that a drinking habit is associated with a lower prevalence of metabolic syndrome [14]; however, several groups including us have repeatedly published an opposing view that drinking habits of more than 20–30 g per day are associated with a higher prevalence [57–60]. We proposed that the current clinical diagnostic criteria of metabolic syndrome may lead to over-diagnosis of metabolic syndrome. Namely, a drinker suffering from alcohol-related syndrome consisting of multiple morbid conditions including hypertension, hyperlipidemia, and diabetes may be judged as having metabolic syndrome regardless of the development of obesity [61]. Even after such subjects, tentatively designated as those with “metabolic syndrome alcohol (+)”, were excluded, the prevalence of metabolic syndrome in excessive drinkers became statistically equal to that in non-excessive drinkers plus teetotalers. However, ethanol consumption was judged not to contribute to the reduced prevalence of metabolic syndrome [61]. These views are compatible with the present finding, *i.e.*, the beneficial effects of drinking on insulin dynamics have limitations. Notably, such counter-views were reported mainly from Asian groups [57–60]; such effects of ethanol consumption may have ethnic differences.

## 4. Conclusions

The present study supports the repeatedly reported views that ethanol consumption is beneficial for levels of HOMA-IR, HOMA-β, or serum insulin. These observations may be attributed to the improvement of insulin resistance due to ethanol consumption, at least in part. However, before drawing conclusion, we must elucidate overall pictures of insulin/glucose dynamics in which complicated interactions of various factors including insulin secretion by pancreatic β-cells, insulin sensitivity of peripheral tissues and the liver, insulin extraction by the liver, and glucose efflux from the liver are involved. Even if the mechanisms are unclear, the significant observations in the present study were that such probable beneficial effects of ethanol were not applicable to subjects with obesity and/or insulin resistance. Although we must be careful for the limitations due to the present experimental design, such as lacking information on the integrating amount of ethanol consumption, types of alcoholic beverage, precise evaluation of the statuses of liver fibrosis and pancreatic cirrhosis, we believe that a drinking habit cannot be a remedy for improving the morbid conditions of such subjects. Furthermore, they must be careful of the adverse effects of ethanol consumption which aggravate the conditions of hypertension, diabetes, and hyperlipidemia. Arising questions that need to be answered are whether ethnic differences are involved in the present features, and whether abstinence is beneficial among drinkers who are suffering from metabolic syndrome.

## Figures and Tables

**Table 1:** Subjects’ profiles [median (range)].

	All subjects (n = 371)	Non-obese subjects* (n = 301)	Obese subjects** (n = 70)	P-value***
Age (year)	48 (30–65)	47 (30–65)	48 (30–65)	0.3296
BMI (kg/m^2^)	22.8 (15.9–32,2)	22.5 (15.9–24.9)	26.1 (25.0–32.2)	<0.0001
SBP (mmHg)	121 (82–181)	119 (82–176)	130 (93–181)	<0.0001
DBP (mmHg)	76 (39–121)	74 (51–111)	81 (52–121)	<00001
TG (mg/dL)	97 (26–677)	92 (26–539)	129 (36–677)	<0.0001
HDLC (mg/dL)	54 (33–111)	55 (33–111)	49 (36–88)	<0.0001
LDLC (mg/dL)	124 (51–213)	125 (51–213)	125 (58–195)	0.5865
FBS (mg/dL)	91 (60–100)	90 (60–100)	93 (79–100)	0.0384
IRI (μIU/μL)	4.5 (1.0–26.0)	4.0 (1.0–24.0)	6.0 (1.0–26.0)	<0.0001
HOMA-IR (μIU·mg/μL·dL)	0.998 (0.195–6.277)	0.948 (0.220–5.748)	1.407 (0.195–5.277)	<0.0001
HOMA-β (mg·μL/dL·μIU)	59.19 (12.41–275.29)	55.39 (12.41–254.12)	78.75 (22.50–275.29)	<0.0001
AST (IU/L)	22 (13–71)	22 (13–55)	24 (14–71)	0.0011
ALT (IU/L)	21 (6–131)	19 (6–88)	28 (10–131)	<0.0001
γGTP (IU/L)	35 (9–1061)	32 (9–1061)	55 (18–256)	<0.0001
Adiponectin (μg/dL)	6.31 (0.38–39.02)	6.45 (0.38–39.02)	5.60 (1.10–24.2)	0.0385

platelet (×10^3^/μL)	241 (119–403)	241 (119–361)	248 (174–403)	0.6887
Averaged number of drinking days (/week)	4 (0–7)	4 (0–7)	5 (0–7)	0.1259

Averaged amount of daily ethanol consumption (g)	20 (0–100)	18 (0–80)	22 (0–100)	0.2818

Ethanol consumption level (g/week)	84 (0–770)	84 (0–770)	87 (0–420)	0.4821

*: subjects with a BMI< = 25.0 kg/m^2^;

**: subjects with a BMI > 25.0 kg/m^2^;

***: comparison between non-obese and obese subjects using Mann Whitney’s U-test; BMI: body mass index; SBP: systolic blood pressure; DBP: diastolic blood pressure; TG: triglycerides; HDLC: high-density lipoprotein cholesterol; LDLC: low-density lipoprotein cholesterol; FBS: fasting blood glucose; HOMA-IR: insulin resistance assessed by the homeostasis model; HOMA-β: pancreatic beta cell function assessed by homeostasis model; AST: aspartate aminotransferase; ALT: alanine aminotransferase; γGTP: gamma glutamyl transpeptidase.

**Table 2:** Relationship between ethanol consumption level and HOMA-IR, serum insulin, or FBS assessed by multiple regression analysis; n = 371, t value (p value).

	*vs.* log HOMA-IR (μIU·mg/μL·dL) (R = 0.601)	*vs* HOMA-β (mg·μL/dL·μIU) (R = 0.581)	*vs.* log serum insulin (μIU/μL) (R = 0.670)	*vs.*FBS (mg/DL) (R = 0.302)
Ethanol consumption level (g/week)	−3.233 (0.0014)	−3.825 (0.0002)	−3.434 (0.0007)	0.778 (0.4368)
Age (year)	−3.651 (0.0003)	−4.597 (<0.0001)	−3.990 (<0.0001)	1.549 (0.1224)
log BMI (Kg/m^2^)	3.819 (0.0002)	3.801 (0.0002)	3.933 (0.0001)	−0.002 (0.9998)
log SBP (mmHg)	1.961 (0.0020)	−0.704 (0.4819)	1.311 (0.1906)	4.781 (0.0001)
log TG (mg/dL)	3.783 (0.0002)	3.951 (<0.0001)	3.668 (0.0001)	0.195 (0.8454)
log HDLC (mg/dL)	−1.267 (0.2061)	−0.906 (0.3653)	−1.328 (0.1850)	0.157 (0.8750)
log LDLC (mg/dL)	3.740 (0.0002)	3.567 (0.0004)	3.897 (0.0100)	−0.300 (0.8192)
log ALT (IU/L)	2.349 (0.0112)	2.375 (0.0181)	2.591 (0.0001)	0.229 (0.8192)
log adiponectin (μg/dL)	−1.409 (0.1596	−1.175 (0.2408)	−1.369 (0.1635)	−0.372 (0.7099)

HOMA-IR: insulin resistance assessed by homeostasis model; FBS: fasting blood sugar; BMI: body mass index; SBP: systolic blood pressure; TG: triglycerides; HDLC: high-density lipoprotein cholesterol; LDLC: low-density lipoprotein cholesterol.

**Table 3:** Effects of BMI on relationship between levels of ethanol consumption and HOMA-IR, HOMA-β, insulin and FBS assessed by multiple regression analyses; t value (p value).

*vs.* ethanol consumption level (g/week)	non-obese subjects* (n = 301)	obese subjects** (n = 70)
log HOMA-IR (μIU·mg/μL·dL)***	−3.159 (R = 0.562, p = 0.0018)	−0.396 (R = 0.677, p = 0.6935)
log HOMA-β (mg·μL/dL·μIU)***	−3.746 (R = 0.536, p = 0.0002)	−0.445 (R = 0.660, p = 0.6578)
log serum insulin (μIU/μL)***	−3.253 (R = 0.537, p = 0.0013)	−0.420 (R = 0.682, p = 0.6759)
log FBS (mg/dL)***	1.057 (R = 0.329, p = 0.2914)	0.078 (R = 0.040, p = 0.9383)

*: subjects with BMIs ≤ 25.0 kg/m^2^;

**: subjects with BMIs > 25.0 kg/m^2^;

***: adjusted by age, log BMI, log SBP, log TG, log HDLC, log LDLC, log ALT and log adiponectin in multiple regression analysis models; BMI: body mass index, HOMA-IR: insulin resistance assessed by homeostasis model; HOMA-β: pancreatic beta cell function assessed by homeostasis mode; FBS: fasting blood sugar.

**Table 4:** Effects of HOMA-IR on relationship between levels of ethanol consumption and HOMA-IR, HOMA-β, insulin and FBS assessed by multiple regression analyses; t value (p value).

*vs.* ethanol consumption level (g/week)	non-insulin resistant subjects* (n = 337)	insulin resistant subjects** (n = 34)
log HOMA-IR (μIU·mg/μL·dL)***	−3.323 (R = 0.601, p = 0.0014)	−1.010 (R = 0.526, p = 0.3222)
log HOMA-β (mg μL/dL·μIU)***	−3.972 (R = 0.508, p < 0.0001)	−0.902 (R = 0.495, p = 0.3755)
log serum insulin (μIU/μL)***	−3.579 (R = 0.521, p = 0.0004)	−1.018 (R = 0.526, p = 0.3183)
log FBS (mg/dL)***	1.033 (R = 0.309, p = 0.3024)	−0.070 (R = 0.382, p = 0.9450)

*: subjects with HOMA-IRs ≤ 25.0 kg/m^2^;

**: subjects with HOMA-IRs > 25.0 kg/m^2^;

***: adjusted by age, log BMI, log SBP, log TG, log HDLC, log LDLC, log ALT and log adiponectin in multiple regression analysis models; BMI: body mass index, HOMA-IR: insulin resistance assessed by homeostasis model; HOMA-β: pancreatic beta cell function assessed by homeostasis mode; FBS: fasting blood sugar.

**Table 5:** Relationship between levels of serum adiponectin and ethanol consumption assessed by multiple regression analyses in subjects without insulin resistance or obesity; t value (p value).

*Vs.* adiponectin (μg/μL)	Subjects with BMIs of 25.0 kg/m^2^ or less (n = 301, R = 0.491)	Subjects with HOMA-IR of 2.0 μIU·g/μL·dL or less (n = 337, R = 0.435)
Ethanol consumption level (g/week)	0.185 (0.2371)	0.675 (0.4999)
Age (year)	0.223 (0.8234)	0.868 (0.3860)
log BMI (Kg/m^2^)	−2.970 (0.0032)	−1.995 (0.0496)
log SBP (mmHg)	−0.371 (0.7106)	−0.460 (0.3208)
log TG (mg/dL)	−1.523 (0.1290)	−0.994 (0.3208)
log HDLC (mg/DL)	4.090 (<0.0001)	4.721 (<0.0001)
log LDLC (mg/dL)	−1.672 (0.0956)	−1.106 (0.2695)
log ALT (IU/L)	−0.425 (0.6709)	−0.716 (0.4747)
log HOMA-IR (μIU·g/μL·dL)	−1.682 (0.0936)	−1.393 (0.1646)

HOMA-IR: insulin resistance assessed by homeostasis model; FBS: fasting blood sugar BMI: body mass index; SBP: systolic blood pressure; TG: triglycerides; HDLC: high-density lipoprotein cholesterol; LDLC: low-density lipoprotein cholesterol.
